# Hemostatic Antimicrobial Hydrogels Based on Silicon, Iron, Zinc, and Boron Glycerolates for Wound Healing Applications

**DOI:** 10.3390/gels10120795

**Published:** 2024-12-05

**Authors:** Tat’yana Khonina, Semyon Alekseenko, Elena Shadrina, Il’ya Ganebnykh, Alexander Mekhaev, Leonid Larionov, Maria Dobrinskaya, Nadezhda Izmozherova, Irina Antropova, Maxim Karabanalov, Muza Kokhan, Natali’ya Evstigneeva, Oleg Chupakhin

**Affiliations:** 1 Postovsky Institute of Organic Synthesis, Ural Branch of the Russian Academy of Sciences, 620108 Ekaterinburg, Russia; sema_alekseenko_1999@mail.ru (S.A.); elenavlad.shadrina@gmail.com (E.S.); ing@ios.uran.ru (I.G.); mehaev@ios.uran.ru (A.M.); ochupakhin@gmail.com (O.C.); 2 Soil Science, Agroecology and Chemistry Department, Ural State Agrarian University, 620075 Ekaterinburg, Russia; 3 Pharmacology and Clinical Pharmacology Department, Ural State Medical University, Ministry of Health of the Russian Federation, 620028 Ekaterinburg, Russia; leonid-larionov@mail.ru (L.L.); maria-nd@mail.ru (M.D.); nadezhda_izm@mail.ru (N.I.); aip.hemolab@mail.ru (I.A.); 4 Institute of New Materials and Technologies, Ural Federal University, 620062 Ekaterinburg, Russia; m.s.karabanalov@urfu.ru; 5 Ural Research Institute for Dermatology, Venereology and Immunopathology, 620076 Ekaterinburg, Russia; mkokhan@yandex.ru (M.K.); evstigneeva-np@yandex.ru (N.E.)

**Keywords:** sol–gel processing, silicon, iron(III), zinc, boron glycerolates, nanoscale structure, glycerohydrogel, hemostatic, antimicrobial and wound healing activity

## Abstract

The use of glycerolates of biogenic elements as biocompatible precursors in sol–gel synthesis is an innovative direction and opens up new scientific and practical prospects in chemistry and technology of producing practically important biomedical materials, including hemostatic, antimicrobial, and wound healing materials. Using biocompatible precursors, silicon, zinc, boron, and iron glycerolates, new bioactive nanocomposite hydrogels were obtained by the sol–gel method. The composition and structural features of the hydrogels were studied using a complex of modern analytical techniques, including TEM, XRD, AES, and ESI MS. Hemostatic activity of the hydrogels was studied in the in vivo experiments; using the example of silicon-iron-zinc-boron glycerolates hydrogel, primary toxicological studies were carried out. Antimicrobial properties of hydrogels were studied using the agar diffusion method. The structural features of hydrogels and their relationship to medical and biological properties were revealed. It was shown that glycerolates hydrogels are non-toxic, and exhibit pronounced hemostatic activity, generally comparable to the commercial hemostatic drug Capramine. Antimicrobial activity is more pronounced for silicon-iron-zinc-boron and silicon-iron-boron glycerolates gel. The results obtained indicate that these glycerolates hydrogels are potential hemostatic and antibiotic-independent antimicrobial agents for topical wound healing applications in medical and veterinary practice.

## 1. Introduction

Today, uncontrolled bleeding remains a major problem in emergency care, surgery, and on the battlefield, as uncontrolled bleeding is the leading cause of premature death in combat trauma and a secondary cause of death in civilian injuries [1,2]. Therefore, highly efficient hemostatic agents play an important role in stopping bleeding and reducing mortality [3,4].

Wound healing is a complex and orderly physiological process that includes four overlapping phases: hemostasis (blood clotting, stopping bleeding), inflammation (suppression of microbial growth and preparation of the wound bed), cell proliferation (stimulation of fibroblast proliferation and their migration to cover the wound surface), and matrix remodeling (tissue strengthening and collagen synthesis) [5,6]. The first phase of wound healing, namely hemostasis, involves the combined action of blood vessels, platelets, and the coagulation system to maintain a closed circulatory system and protect the body from severe blood loss [4]. To achieve hemostasis, different types of hemostatic materials are used, each of which has its own mechanism of action and is used depending on the type of injury, the severity of bleeding, the size and configuration of the wound, its location on the body, accessibility to the bleeding site, and the patient’s coagulation function [1].

There are many options for stopping externally accessible bleeding, such as gauze, dressings, sprays, granules, powders, adhesives, hydrogels, tourniquets, or physical devices [1,3,4,7,8,9,10]. These products are generally made from natural polymers (e.g., chitosan, gelatin, collagen, cellulose, and alginate), synthetic polymers (e.g., poly(cyanoacrylate), poly(acrylic acid), and polylactic acid), or inorganic silicon compounds (e.g., kaolin and zeolites) [4]. At the same time, natural polymers are highly biocompatible, but possess insufficient mechanical strength, while synthetic polymers have strong mechanical properties, but cannot meet biocompatibility requirements. None of the modern hemostatic agents meet all the requirements of ideal hemostasis.

It is believed that the use of hydrogels is a good strategy to achieve hemostasis in both internal and external bleeding [11]. A common characteristic of hydrogels used to stop external bleeding is adhesion, which allows hydrogels to seal the wound and accelerate hemostasis [9]. Adhesion is mainly due to the reaction between the active groups of hydrogels and the amino groups of the proteins of native tissues. When hydrogel materials act on superficial wounds, the surface charge and other components of the materials directly activate platelets and promote the formation and aggregation of red blood cells, which ultimately leads to coagulation, i.e., thrombus formation [2,7,9,12]. At the same time, hemostatic hydrogels provide a moist environment at the wound site thereby stimulating tissue regeneration and promoting wound healing once hemostasis is achieved [11]. Hydrogels can also absorb inflammatory exudate from the wound, provide a breathable microenvironment, prevent the colonization of anaerobic bacteria, and release preloaded therapeutic agents [5,11,13,14,15,16].

An ideal hemostatic hydrogel should generally have rapid and sustained hemostatic efficacy, biocompatibility, biodegradability, non-cytotoxicity, strong adhesion in a moist environment, and the ability to be gently removed or biodegradable, as well as prevent infection. In addition, ease of use, shelf life, and cost are also important factors to consider when designing and developing modern hemostatic materials [2,3]. It should be noted that only polymeric organic gels based on natural polymers of plant and animal origin or synthetic polymers are considered, as a rule, in the review literature on hemostatic and wound healing hydrogels [17]. Information on inorganic hydrogels for biomedical use, such as colloidal silica hydrogel obtained by the sol–gel method, is presented to a much lesser extent [14,18].

The functionality of polymer hydrogels can be significantly expanded through the process of their biomimetic mineralization [11,19,20,21], as well as the introduction of nanomaterials [3,4,6,11,13,22,23]. In the process of the biomimetic mineralization of polysaccharides, proteins, and synthetic biopolymers, especially by silicon alkoxides or silicates, hybrid materials with a unique structure and set of new useful properties are formed [24,25,26]. The introduction of nanomaterials, which represent a large category of materials with diverse functionalities, can also endow hydrogels with additional and multiple functions to meet the needs for the complex characteristics of hemostatic and wound healing agents, such as antimicrobial properties, mechanical strength, structural stability, etc. [3,4,6,11,13,22].

Injured tissues are susceptible to bacterial infections, so much attention is paid to the combination of hemostatic and antibacterial activity [7,8]. It should be noted that, due to the increasing prevalence of drug-resistant bacterial infections and the slow healing of chronically infected wounds, the development of new antimicrobial agents with different action mechanisms excluding and/or minimizing the possibility of formation of resistant strains has become a serious problem [6].

One of the methods for producing hybrid biomimetic and nanocomposite hydrogels is the sol–gel method [27,28,29]. The classic sol–gel method is a physicochemical process based on hydrolysis and condensation reactions in the precursor solution leading to the formation of a new phase. These chemical reactions are closely connected with the physical colloidal transformations, which lead to the formation of a gel or a precipitate [30,31]. At the same time for alkoxy precursors traditionally used in sol–gel synthesis, in particular, silicon and titanium alkoxides, the mechanism of sol–gel transformations, the influence of various factors on these transformations, as well as on the composition and structure of the resulting products, are quite well described in the literature.

A promising direction in sol–gel chemistry is the use of element-containing derivatives of polyhydric alcohols as biocompatible precursors [32,33,34,35,36,37,38,39,40,41,42]. Their use for biomedical purposes is preferable, since the polyol released in the hydrolysis and condensation reactions does not adversely affect biomacromolecules, as for example, monohydric alcohols, which cause their denaturation and/or precipitation. In addition, such sol–gel processing runs under the mild conditions, as a rule, at room temperature, without the use of any catalysts or organic solvents, and thus can be related to the green chemistry methods that seem promising for biomedical applications. It should also be noted that in this case, as a rule, transparent monolithic hydrogels are formed, resistant to syneresis [33,35,37]. Polyolates precursors based on ethylene glycol, 1,2-propanediol, glycerol, and polyethylene glycol were used for the biopolymers and protein molecules immobilization, for the porous monolithic materials production, and in bioaffinity chromatography, as well as for biocatalysts, biosensors, and drug delivery systems production [26].

In a recent review [26], the advantages and disadvantages of currently known precursors based on ethylene glycol, 1,2-propanediol, glycerol, polyethylene glycol, and protocols proposed for the immobilization of biopolymers and cells, as well as for sol–gel synthesis of nanocomposites, are critically analyzed. In this case, preference is given to the liquid derivative of ethylene glycol—individual tetrakis (2-hydroxyethyl)orthosilicate. However, ethylene glycol has a negative effect on living cells and is not approved for medical use, which limits its use.

We have previously developed methodological approaches to the sol–gel synthesis of pharmacologically active hydrogels for topical applications [37,38]. The essence of the methodology lies in the use of biogenic elements polyolates, primarily silicon polyolates, as biocompatible precursors in sol–gel processing. Element-containing polyolates are used in excess of the corresponding polyols and are approved for use in medicine (glycerol, 1,2-propanediol, polyethylene glycol-400), which has a significant effect on the gelation process, composition, and structure of the resulting products. Within the framework of the developed methodological approaches, on the basis of Si, Zn, B, and Fe glycerolates, we synthesized a number of hydrogels including nanocomposite ones, with complexes of useful pharmacological properties. The use of silicon glycerolates gives the gels reparative and regenerative properties—zinc, boron, and iron glycerolates have immunotropic, antimicrobial, and hemostatic properties, respectively [43,44,45]. It should be noted that all the gels synthesized by us can be used both as independent medicinal agents for topical application, and as the basis of pharmaceutical compositions that ensure the efficient delivery of drug additives in the treatment of diseases of the skin, soft tissues, and mucous membranes of various etiologies [46].

Recently synthesized by us, Si-Fe–glycerolates hydrogel [46,47] exhibits pronounced hemostatic and wound healing activity, especially in the presence of chitosan; when dispersed, it turns into an ointment-like state, is easily distributed on the skin and mucous membranes, and is of practical interest for the treatment of skin and soft tissues traumatic injuries. However, this gel does not exhibit antimicrobial activity. Since injured tissues are susceptible to bacterial infections, hydrogels should have both hemostatic and antimicrobial effects, which is also necessary in the treatment of infected wounds. We assume that modification of the Si-Fe–glycerolates hydrogel with zinc and boron glycerolates will lead to the appearance of antimicrobial activity along with hemostatic and wound healing effects.

The purpose of this work was the sol–gel synthesis and characterization of new nanocomposite hydrogels obtained using silicon, iron, zinc, and boron glycerolates as precursors, studying the hemostatic and antimicrobial effects, establishing the relationship with structural features, as well as conducting primary toxicological studies (in vivo).

## 2. Results and Discussion

### 2.1. Characteristics of Glycerolates Precursors

**Figure 1:**
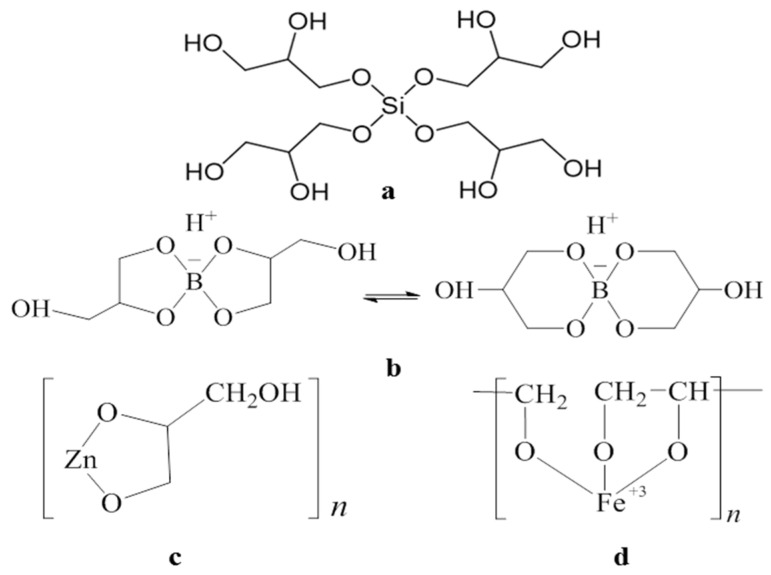
The biocompatible precursors used in the sol–gel synthesis of the glycerolates hydrogels: (**a**) silicon tetraglycerolate, (**b**) boron bisglycerolates, (**c**) zinc monoglycerolate, and (**d**) iron(III) monoglycerolate.

The four biocompatible precursors used in the sol–gel synthesis of the new glycerolates hydrogels are presented in Figure 1.

It is known that silicon tetraglycerolate Si(C_3_H_7_O_3_)_4_ (Figure 1a), due to its polyfunctional character, tends to form oligomeric species via an equilibrium intermolecular condensation reaction. Therefore, its synthesis was carried out in an excess of glycerol that minimized possible condensation processes [37]; the resulting glycerin solution of silicon glycerolates was used in the hydrogel synthesis.

According to ^11^B NMR spectral data [48], liquid boron bisglycerolates H[B(C_3_H_6_O_3_)_2_] exist as a mixture of two bicyclic structural isomers (Figure 1b) possessing acid properties. Unlike Si(C_3_H_7_O_3_)_4_, H[B(C_3_H_6_O_3_)_2_] were synthesized and used without an excess of glycerol [44].

Zinc monoglycerolate ZnC_3_H_6_O_3_ [49,50] was synthesized in an excess of glycerol, and then the resulting colloidal suspension was used to prepare glycerohydrogels [43,44].

Iron(III) monoglycerolate FeC_3_H_5_O_3_ (Figure 1d) is an individual crystalline substance, received by us for the first time [51]. When dispersed in glycerol, it forms a colloidal suspension [45], as in the case of ZnC_3_H_6_O_3_ [43]. Moreover, in both cases, the TEM method showed the amorphous nature of glycerolates in a colloidal suspension.

It is known that Si(C_3_H_7_O_3_)_4_ and H[B(C_3_H_6_O_3_)_2_] are readily hydrolyzed in an aqueous medium [37,44], whereas ZnC_3_H_6_O_3_ is more resistant to hydrolytic transformations [43,50]. FeC_3_H_5_O_3_ is more resistant to hydrolysis. As shown in our study [45], in this case, hydrolysis proceeds to a much lesser extent, and the addition of glycerol slows down the process. Thus, XRD and elemental analysis showed that the hydrolysis of 0.1% FeC_3_H_5_O_3_ in water, which proceeds by ~10% in 14 days, is completely suppressed in an aqueous glycerol media containing 30% glycerol. For comparison, in the case of ZnC_3_H_6_O_3_, during the same time in an aqueous glycerol medium, hydrolysis proceeds for 40% [43].

### 2.2. Preparation of Glycerohydrogels 

Hydrogels based on glycerolates of silicon, iron, and zinc (Si-Fe-Zn–gel), silicon, iron, and boron (Si-Fe-B–gel), and silicon, iron, zinc, and boron (Si-Fe-Zn-B–gel) were prepared using the corresponding glycerolates by the sol–gel method in an aqueous glycerol medium (molar ratios of the initial substances are presented in Table 1). The gels were obtained at 70 °C; the gelation time (the time from the start of the initial substances mixing to the complete disappearance of the system fluidity) varied from 15 min to 9 h. The molar ratio of the initial substances was optimal in terms of pharmaco-technological characteristics (storage stability, required consistency, easy and nontraumatic application to skin and mucous membranes, and pharmacological efficacy).

The gels are light green semisolid substances that are stable toward syneresis; they are not soluble in common organic solvents and are partially soluble in water; and the gels are easily transformed to a grease-like condition, well distributed into the skin and mucosa if dispersed, and can be easily rinsed with a 0.9% sodium chloride solution.

### 2.3. Rheological Properties

One of the main criteria when developing bioactive gel compositions is their rheological characteristics. It was established that all glycerohydrogels studied have thixotropic properties characteristic for non-Newtonian liquids; their viscosity depends on the shear stress, and the flow rheogram is nonlinear [18]. In this case, the experimentally determined values of the effective dynamic viscosity of glycerohydrogels are 2.5–11.8 Pa∙s over a range of shear rates 10–400 s^−1^. This viscosity range provides optimal operational (consumer) characteristics: ease, uniformity, and non-traumatic application to damaged areas of the skin or mucous membrane, and corresponds to those given in the work [52] of less than 14 Pa∙s.

### 2.4. FTIR Analysis

**Figure 2:**
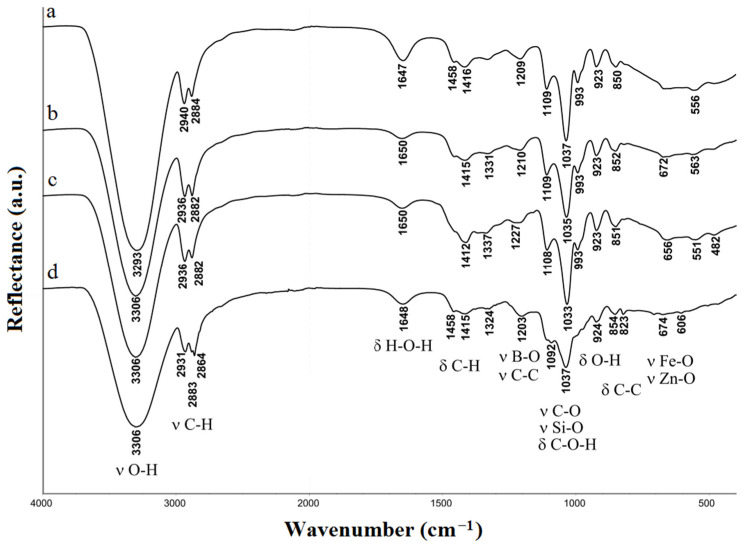
IR spectra of (**a**) Si-Fe– (for comparison), (**b**) Si-Fe-Zn–, (**c**) Si-Fe-B–, and (**d**) Si-Fe-Zn-B–gel.

The absorbance bands of O–H, C–H, Si–O, B–O, Zn–O, Fe–O, and H–O–H species in the gels were probed by FTIR analysis (Figure 2).

As can be seen from Figure 2, there are no noticeable differences in spectra of the samples. The broadened bands at 3293–3306, 1647–1650, and 923 cm^−1^ indicate the presence of water molecules in glycerohydrogels, and also OH groups in glycerol and glycerol residues. Intense bands at 2864–2940 cm^−1^ in the spectra correspond to the stretching vibrations of C–H bonds, and bands with maxima in the range of 1324–1458 cm^−1^ correspond to the bending vibrations of C–H bonds (wagging, twisting, and scissoring vibrations) in the CH and CH_2_ of glyceroxy groups. Weak broadened bands in areas 1209–1227 and 823–854 cm^−1^ correspond to the stretching and bending vibrations of bonds C–C. The spectra of gels also contain broadened absorption bands in the region of 993–1109 cm^−1^, which are characteristic of the silicon glycerolates (the bands at 993, 1033, 1037, 1092, and 1109 cm^−1^, corresponding to both C–O stretching vibrations in C–O–H of the glycerol residue, as well as Si–O and Si–O–Si).

In addition, the spectra of Si-Fe-Zn– and Si-Fe-Zn-B–gel contain low-intensity absorption bands in the region 482–674 cm^−1^, corresponding to bond stretching vibrations Fe–O and Zn–O. Spectra Si-Fe-B– and Si-Fe-Zn-B–gel also contain absorption bands in the range 1200–1230 cm^−1^, corresponding to the B–O stretching vibrations in the Si–O–B grouping.

### 2.5. TEM Analysis

**Figure 3:**
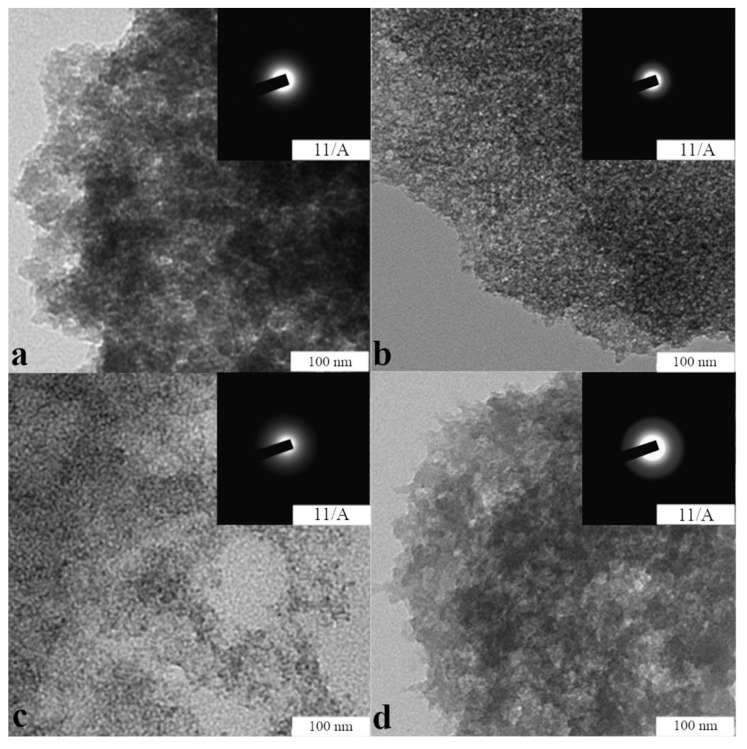
TEM micrographs of dried suspension: (**a**) Si-Fe–(for comparison), (**b**) Si-Fe-Zn–, (**c**) Si-Fe-B–, (**d**) Si-Fe-Zn-B–gel in ethanol. (**a**–**d**) High-resolution TEM image, inserts show electron diffraction area.

Figure 3 shows TEM micrographs of Si-Fe–, Si-Fe-Zn–, Si-Fe-B–, and Si-Fe-Zn-B–gel applied as a thin layer on a copper grid and dried in vacuo.

Figure 3 reveals the presence of (a–d) nanoscale particles in the high-resolution region and absence of point and ring reflexes in the field of electron diffraction, which are characteristic for crystalline substances, namely the starting FeC_3_H_5_O_3_ and ZnC_3_H_6_O_3_ substances. Thus, the received TEM micrographs indicate that in multicomponent heterogeneous systems, which are the studied gels, there are no crystalline components similar to [44].

### 2.6. TGA

**Figure 4:**
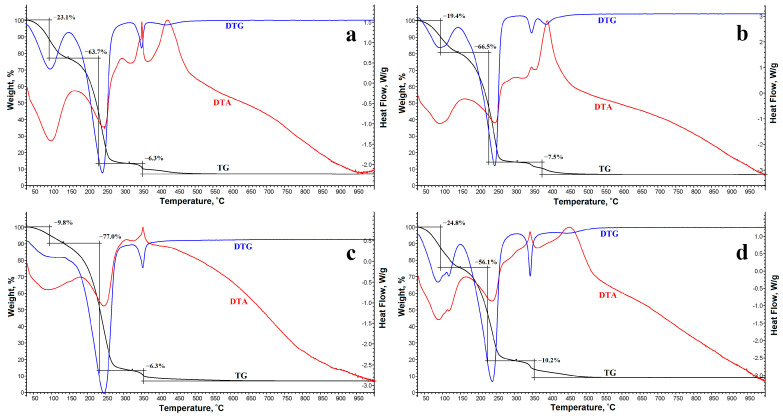
Thermal analysis data for (**a**) Si-Fe–, (**b**) Si-Fe-Zn–, (**c**) Si-Fe-B–, (**d**) Si-Fe-Zn-B–gel.

The thermal stability of Si-Fe–, Si-Fe-Zn–, Si-Fe-B–, and Si-Fe-Zn-B–gel was studied by thermogravimetric analysis (TGA) and differential thermogravimetric analysis (DTG) (Figure 4).

For all synthesized gel samples, it can be seen from the TG curves that the thermal decomposition process occurs by three steps of weight loss (Table 2). At the first step, at temperatures up to ~140–150 °C, water predominantly evaporates from the gels. At the second step of decomposition at a temperature of 150 to 320 °C, glycerol evaporates and silicon glycerolates and glycerol decompose. Above 320 °C, the decomposition of iron and zinc glycerolates occurs, accompanied by a pronounced exothermic effect on the DTA curve [45,50].

### 2.7. AES Analysis

Table 3 presents the AES data of elements contained in the gels and their dispersed (solid) phase. The direct analysis of the elements’ content in a dispersion (liquid) medium confirmed the reliability of their determinations in the solid phase.

According to the AES data, the main amount of silicon (75–80%) was determined in the extracted dispersed (solid) phases, and the remaining content of silicon was determined in the liquid dispersion mediums. Practically all iron contained in the gels was determined in the isolated solid phases (more than 97%). The total amount of zinc contained in the Si-Fe-Zn–gel was also determined in the solid phase, but the solid phase of Si-Fe-Zn-B–gel contained only 78% zinc, and the rest of it was determined in the liquid medium. In the case of Si-Fe-B– and Si-Fe-Zn-B–gel, the main amount of boron (82–94%) was determined in the liquid mediums.

Based on the data obtained (AES and ESI MS, see below), it can be assumed that the liquid Si-Fe-Zn-B–gel medium, as in the case of Si-Zn-B–gel characterized by us earlier, contains in its composition water-soluble zinc bisglyceroborates Zn[B(C_3_H_6_O_3_)_2_]_2_ [44].

### 2.8. ESI MS Analysis

**Figure 5:**
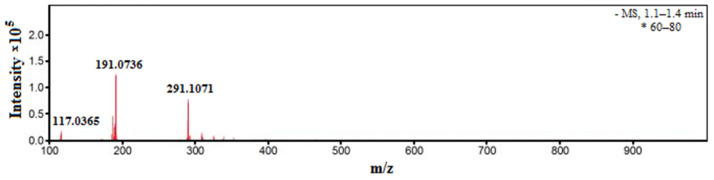
ESI mass spectrum in negative mode of Si-Fe-Zn-B–gel liquid medium (* averaged for scan number from 60 to 80).

Figure 5 shows ESI mass spectrum in the negative mode of Si-Fe-Zn-B–gel liquid medium.

The ESI mass spectrum in the negative mode of the liquid medium of Si-Fe-Zn-B–gel was characterized by the presence of a strong peak at *m*/*z* 191, attributable to the [B(C_3_H_6_O_3_)_2_]^−^ anion [48]. The appearance of weak peak at *m*/*z* 117 is probably associated with the formation of HOB(C_3_H_6_O_3_), the product of hydrolysis of H[B(C_3_H_6_O_3_)_2_], and peak at 291, with the formation of H[B(C_3_H_6_O_3_)]_2_(C_3_H_5_O_3_), which is the condensation product of HOB(C_3_H_6_O_3_) and H[B(C_3_H_6_O_3_)_2_] [44]. In our opinion, the formation of iron(III) trisglyceroborates Fe[B(C_3_H_6_O_3_)_2_]_3_ is unlikely, which, according to the mechanism, may be due to the greater resistance to hydrolysis of FeC_3_H_5_O_3_ compared to ZnC_3_H_6_O_3_.

### 2.9. XRD Analysis

**Figure 6:**
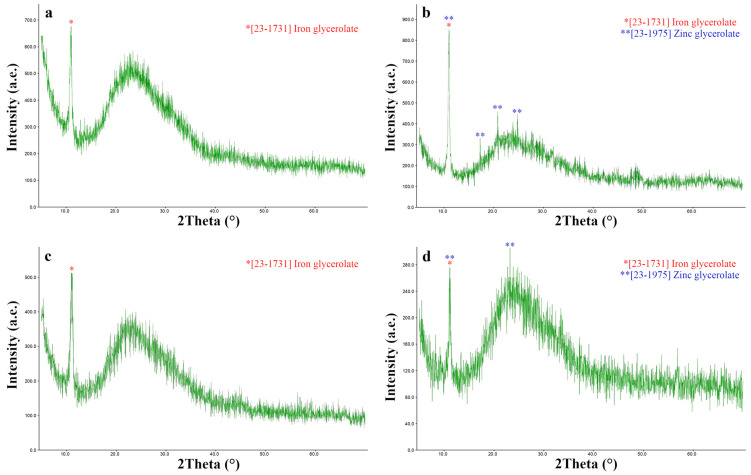
XRD patterns of extracted solid phase of (**a**) Si-Fe– (for comparison), (**b**) Si-Fe-Zn–gel, (**c**) Si-Fe-B–gel, and (**d**) Si-Fe-Zn-B–gel.

Figure 6 shows the XRD patterns of the extracted solid phases of Si-Fe, Si-Fe-Zn–, Si-Fe-B–, and Si-Fe-Zn-B–gel.

The XRD patterns correspond to a heterophase product, which is an amorphous component at the angle interval (15 < 2θ < 35), which is a 3D silicone polymeric gel network [47]. Peak at 12.7 deg. 2θ (8.1 Å) is the main diffraction line of FeC_3_H_5_O_3_ and corresponds to the data [53]. In addition, the X-ray diffraction pattern solid phases of the Si-Fe-Zn– and Si-Fe-Zn-B–gel contains the same main diffraction line, corresponding to crystalline ZnC_3_H_6_O_3_ [50].

The presence of an organic component (residual glyceroxy groups at silicon, iron, and zinc atoms) in the extracted dispersed phase of the hydrogels was demonstrated in IR spectroscopy, as was evidenced by the absorption bands (ν/cm^−1^): 3306 (OH), 2936, 2882 (C–H, CH_2_), 1459–1208 (C–H, CH_2_), and 1109–656 (C–O, Si–O, C–O–Si, Si–O–Si, C–O–Fe, Fe–O, Zn–O). The elemental analysis data also confirmed the presence of an organic component in the composition of the gels’ dispersed phases (C, 15–17%; H, 3–4%).

It should be noted that when the dispersed phase is extracted, the crystallization of amorphous iron and zinc glycerolates occurs, as evidenced by XRD data, similar to [43].

Thus, all studied gels are products of sol–gel transformations: hydrolysis o precursors (primarily Si(C_3_H_7_O_3_)_4_ and H[B(C_3_H_6_O_3_)_2_]) and subsequent condensation, results in the formation of a 3D framework of hydrogels containing mainly Si-O-Si groupings with residual glyceroxy groups at the silicon atom [44]. Other precursors such as ZnC_3_H_6_O_3_ and FeC_3_H_5_O_3_ (to a lesser extent) also undergo partial hydrolysis and are located in the cells of the 3D framework in a nano-sized amorphous state; when the solid phase of the gels is released, they crystallize.

The dispersion medium of the gels is a water–glycerol solution of primarily Si(C_3_H_7_O_3_)_4_ and H[B(C_3_H_6_O_3_)_2_] (for Si-Fe-B– and Si-Fe-Zn-B–gel), which also contains water-soluble products of their hydrolytic transformations. In addition, the Si-Fe-Zn-B–gel dispersion medium contains water-soluble zinc bisglyceroborates Zn[B(C_3_H_6_O_3_)_2_]_2_.

### 2.10. Pharmacological Studies

#### 2.10.1. Toxicological Profile of Glycerohydrogels

The toxicological profile study included skin-resorptive, skin-irritant, local irritant, and sensitizing effects, which was carried out using Si-Fe-Zn-B–gel, containing four biocompatible precursors (Si(C_3_H_7_O_3_)_4_, FeC_3_H_5_O_3_, ZnC_3_H_6_O_3,_ H[B(C_3_H_6_O_3_)_2_]), in accordance with the Guidelines for Preclinical Studies [54] and our previous research [46].

**Figure 7:**
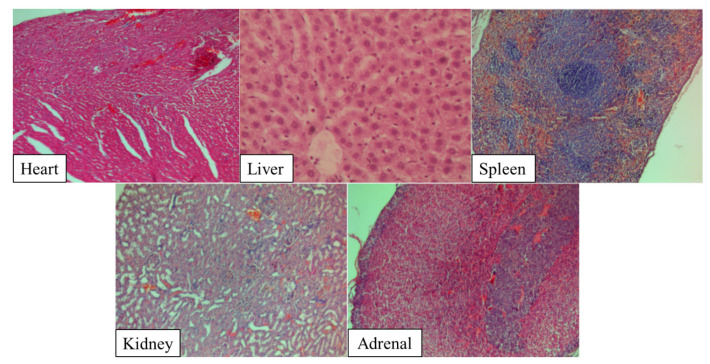
Histological analysis of Si-Fe-Zn-B–gel treated group 14 days after administration, hematoxylin-eosin, magnification ×100.

When assessing the condition of laboratory animals after testing, animal death and adverse clinical signs were not recorded in any group of animals during the entire observation period. Epicutaneous application of the studied samples did not lead to any reactive disturbances both during the first five hours after application and in the next 14 days. When assessing the irritating effects and properties of Si-Fe-Zn-B–gel using the conjunctival test and sensitizing effect, no deviations in the studied parameters were detected during the entire observation period. Histological studies did not reveal any abnormalities in all tissue samples (heart, liver, spleen, kidney, adrenal) in the test and control groups 14 days after hydrogel administration. There were no inflammatory, allergic cellular reactions or significant structural changes (Figure 7).

Thus, the newly synthesized Si-Fe-Zn-B–gel offers a promising direction, as previous studies have shown [55].

#### 2.10.2. Hemostatic Properties of Glycerolates Hydrogels

**Figure 8:**
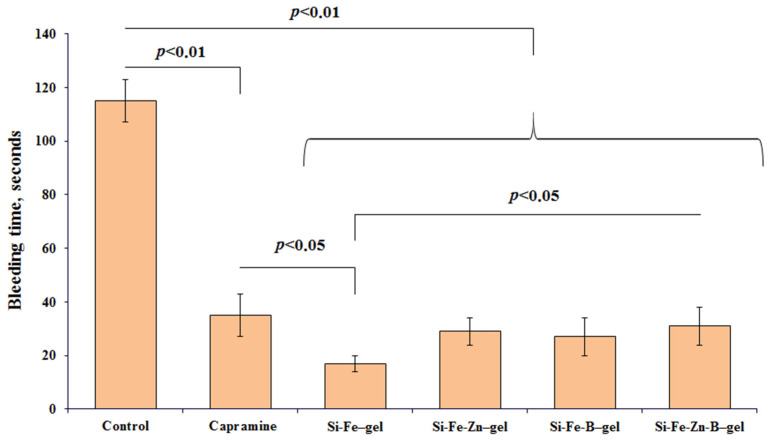
Comparative assessment of bleeding time in mice with incised liver wounds.

According to the literature data [54,56,57], the most reliable hemostatic effect results of the substances are obtained by capillary parenchymal bleeding modeling (in vivo) on the liver, spleen, and kidneys of experimental animals. Figure 8 shows the results of Si-Fe–, Si-Fe-Zn–, Si-Fe-B–, and Si-Fe-Zn-B–gel hemostatic activity studies using the liver wound model in mice, in comparison to the control (without the use of drugs) and the commercial hemostatic agent Capramine, the main active ingredient of which is iron(III) sulfate [58,59].

As follows from Figure 8, for all tested gels, the duration of bleeding from a liver wound was lower compared to the control group (*p* < 0.01). Si-Fe-Zn–, Si-Fe-B–, and Si-Fe-Zn-B–gel practically did not differ in activity from each other and were not inferior to the comparison drug Capramine. At the same time, the increased hemostatic activity of Si-Fe–gel compared to other glycerolates hydrogels and Capramine (*p* < 0.05) may be associated with a higher, approximately 2-fold, content of Fe (Table 3) and therefore the increased content of FeC_3_H_5_O_3_. It should be noted that one of the advantages of the resulting gels, compared to Capramine, is a wound-healing and antimicrobial effect, which is not typical for Capramine.

It can be assumed that the hemostatic effect of gels is determined mainly by nano-sized particles of FeC_3_H_5_O_3_, where the mechanism of action is probably similar to iron oxide nanoparticles and is associated with platelet activation and erythrocyte aggregation [60].

#### 2.10.3. Antimicrobial Properties of Glycerolates Hydrogels

The results of studying the antimicrobial activity of gels using the agar diffusion method [44,54,61] are shown in Table 4.

**Figure 9:**
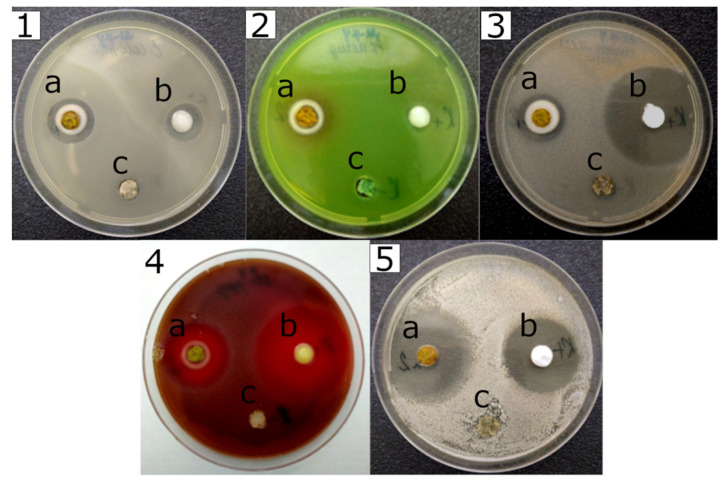
Strain growth inhibition zones: (**1**) *E. coli* ATCC 8739; (**2**) *P. aeruginosa* ATCC 9027; (**3**) clinical strain *S. aureus* (MRSA); (**4**) *S. pyogenes* ATCC 19615. (**5**) *C. albicans* RCPF _Y_-401/NCTC-885-653: (**a**) Si-Fe-Zn-B-gel; (**b**) positive control; (**c**) silicon glycerolates gel (negative control).

As can be seen from Table 4, Fe-Si–gel has weak antibacterial activity against *S. aureus*, including clinical strain MRSA. Si-Fe-Zn–gel has high antibacterial activity against *S. pyogenes* and high fungicidal activity against *C. albicans*. Si-Fe-B–gel has high antibacterial activity against *S. aureus*, including clinical strains, *S. pyogenes*, and moderate effects; in relation to *P. aeruginosa*, it exceeds the positive control. The gel also exhibits high fungicidal activity against *C. albicans*. Si-Fe-Zn-B–gel has high antibacterial activity against *E. coli* and *P. aeruginosa*, exceeding the positive control, as well as *S. pyogenes* and clinical strains *S. aureus* and *S. aureus* (MRSA). In addition, the gel showed high fungicidal activity against *C. albicans*, comparable to the positive control (Figure 9).

Thus, all gels have antimicrobial activity of varying degrees of severity: the most active are Si-Fe-Zn-B– and Si-Fe-B–gel where, in some cases, antimicrobial activity is comparable to or higher than the positive control. It should be noted that the boron content in the Si-Fe-Zn-B–gel is approximately two times less than in the Si-Fe-B–gel (see Table 3), but at the same time, in our opinion, the resulting zinc glyceroborates Zn[B(C_3_H_6_O_3_)_2_]_2_ imparts additional antimicrobial activity to the gel.

## 3. Conclusions

Thus, it can be concluded that Si-Fe-Zn–, Si-Fe-B–, and Si-Fe-Zn-B–gels are typical nanocomposite gels. The polymeric 3D network of the gels is formed as a result of the incomplete hydrolysis of silicon glycerolates because of glycerol excess and subsequent silanol condensation reactions with the formation in the main Si–O–Si groupings. The network contains residual glyceroxy groups at the silicon atoms. The cells of the dispersed phase contain zinc and iron(III) glycerolates and the products of their partial hydrolysis in the form of separate nano-sized phases. In this case, iron(III) monoglycerolate shows a hemostatic effect and zinc monoglycerolate shows a moderate antimicrobial effect.

The dispersion medium of the gels contains partially hydrolyzed, low-molecular-weight silicon glycerolates, which primarily provide its reparative and regenerative activities.

Boron bisglycerolates in a dispersion medium of Si-Fe-B– and Si-Fe-Zn-B–gel easily and reversibly hydrolyze to form boric acid and glycerol, while providing the antimicrobial activity of the gels. Only a small part of boron is detected in the separated dispersed phase, apparently forming Si–O–B groupings in the spatial framework of the gels. In addition, for Si-Fe-Zn-B–gel it is also possible to form water-soluble zinc bisglyceroborates Zn[B(C_3_H_6_O_3_)_2_]_2_, as in the case of Si-Zn-B–gel, described by us earlier; this also increases its antimicrobial activity.

The gels are non-toxic, exhibit hemostatic activity that is not inferior to the reference drug Capramine, while the antimicrobial activity is most pronounced for Si-Fe-Zn-B– and Si-Fe-B–gel. These gels can be easily obtained from available raw materials using green chemistry methods and are of undoubted interest for implementation in medical and veterinary practice.

## 4. Materials and Methods

### 4.1. Materials

Tetraethoxysilane Si(OC_2_H_5_)_4_ (reagent grade, ECOS-1, Moscow, Russia) was distilled at atmospheric pressure; and glycerol C_3_H_8_O_3_ (analytical grade, Vekton, St. Petersburg, Russia) was distilled in vacuo. Boric acid H_3_BO_3_ (analytical grade, Vekton, St. Petersburg, Russia) was purified by recrystallization. Zinc oxide ZnO (analytical grade), iron(III) chloride hexahydrate FeCl_3_·6H_2_O (analytical grade), and sodium hydroxide NaOH (analytical grade) were purchased from Reakhim, Moscow, Russia. Ethanol (95%) was produced by Constanta-Pharm, Moscow, Russia. Water obtained after double distillation was used.

### 4.2. Synthesis of Glycerolate Precursors

#### 4.2.1. Synthesis of Silicon Tetraglycerolate Si(C_3_H_7_O_3_)_4_ in Excess of Glycerol

Silicon tetraglycerolate Si(C_3_H_7_O_3_)_4_ in 3, 4.5, and 8 M excess of glycerol was synthesized by transesterification of Si(OC_2_H_5_)_4_ with an excess of glycerol (130 °C; molar ratio of 1:7, 1:8.5, and 1:12, correspondently), followed by ethanol removal according to ref. [37]. The products obtained (glycerol solutions of silicon glycerolates) were colorless transparent viscous liquids, readily soluble in water. The results of C, H-elemental analysis and IR spectroscopy corresponded to the data reported in [37].

#### 4.2.2. Synthesis of Iron(III) Monoglycerolate FeC_3_H_5_O_3_

Iron(III) monoglycerolate FeC_3_H_5_O_3_ was synthesized similarly to the study [51] by adding iron(III) chloride hexahydrate FeCl_3_·6H_2_O and NaOH to anhydrous glycerol. The product was a green crystal powder insoluble in water and organic solvents. The results of C, H-elemental analysis, IR spectroscopy, XRD, and Mössbauer spectroscopy corresponded to the data reported in [51].

#### 4.2.3. Synthesis of Zinc Monoglycerolate ZnC_3_H_6_O_3_ in 6 M Excess of Glycerol

The synthesis of zinc monoglycerolate in 6 M excess of glycerol was carried out according to refs. [43,44] under heating of ZnO powder in glycerol (130 °C; molar ratio of 1:7). The resulting product was a white viscous liquid (glycerol suspension) easily miscible with water. The composition of the product obtained corresponded to a molar ratio of ZnC_3_H_6_O_3_–C_3_H_8_O_3_ 1:6 and the data reported in [43,44].

#### 4.2.4. Synthesis of Boron Bisglycerolates HB(C_3_H_6_O_3_)_2_

Boron bisglycerolates were synthesized similarly to [44] by heating glycerol and boric acid (110 °C; molar ratio of 2:1), followed by the addition of toluene to remove the resulting water as an azeotrope. A clear viscous liquid was obtained and was highly soluble in glycerol and water. The following was found (%): C, 37.74; H, 6.98; B, 5.32. C_6_H_13_O_6_B. The following was calculated (%): C, 37.54; H, 6.83; B, 5.63. The results of IR spectroscopy and ^11^B NMR spectrometry corresponded to the data reported in [44,48].

### 4.3. Synthesis of Hydrogels

#### 4.3.1. Synthesis of Si-Fe–Gel

Silicon glycerolates in 3M excess of glycerol Si(C_3_H_7_O_3_)_4_·3C_3_H_8_O_3_ (58.43 g, 0.0874 mol) and iron(III) monoglycerolate FeC_3_H_5_O_3_ (3.80 g, 0.0262 mol) were mixed and stirred at room temperature until a homogeneous mixture was formed. Then, H_2_O (37.77 g, 2.0969 mol) was added with vigorous stirring, and stirring continued at 70 °C until the system lost fluidity and a homogeneous light green gel was formed. The yield of the product was 100.00 g (100%). The composition of the product corresponds to a molar ratio of Si(C_3_H_7_O_3_)_4_–FeC_3_H_5_O_3_–C_3_H_8_O_3_–H_2_O 1.00:0.30:3.00:24.00. The resulting product was analyzed using IR spectroscopy and AES (Si, Fe) (Table 5) according to ref. [47].

#### 4.3.2. Synthesis of Si-Fe-Zn–Gel

Silicon glycerolates in 8M excess of glycerol Si(C_3_H_7_O_3_)_4_·8C_3_H_8_O_3_ (57.33 g, 0.0508 mol), iron(III) monoglycerolate FeC_3_H_5_O_3_ (1.84 g, 0.0127 mol), and zinc monoglycerolate in 6M excess of glycerol ZnC_3_H_6_O_3_·6C_3_H_8_O_3_ (17.97 g, 0.0254 mol) were mixed and stirred at room temperature until a homogeneous mixture was formed. Then, H_2_O (22.86 g, 1.2700 mol) was added with vigorous stirring, and stirring continued at 70 °C until the system lost fluidity and a homogeneous light green gel was formed. The yield of the product was 100.00 g (100%). The composition of the product corresponds to a molar ratio of Si(C_3_H_7_O_3_)_4_–FeC_3_H_5_O_3_–ZnC_3_H_6_O_3_–C_3_H_8_O_3_–H_2_O 1.00:0.25:0.50:11.00:25.00. The resulting product was analyzed using IR spectroscopy and AES (Si, Fe, Zn) (Table 5).

IR, ν/cm^−1^: 3300 (OH); 2932, 2881 (C–H, CH_2_, CH); 1645 (H–O–H); 1436, 1418, 1325 (C–H, CH_2_, CH); 1109, 1088, 1040, 992, 922, 855, 817 (C−O, C–C, C–O–Fe, Si−O−C, Si−O−Si); 647 (Zn−O); 560 (Fe–O).

#### 4.3.3. Synthesis of Si-Fe-B–Gel

Silicon glycerolates in 8M excess of glycerol Si(C_3_H_7_O_3_)_4_·8C_3_H_8_O_3_ (62.46 g, 0.0553mol), iron(III) monoglycerolate FeC_3_H_5_O_3_ (2.01 g, 0.0138 mol), and boron bisglycerolates HB(C_3_H_6_O_3_)_2_ (10.62 g, 0.0553 mol) were mixed and stirred at room temperature until a homogeneous mixture was formed. Then, H_2_O (24.91 g, 1.3825 mol) was added with vigorous stirring, and stirring was continued at 70 °C until the system lost fluidity and a while homogeneous light green gel was formed. The yield of the product was 100.00 g (100%). The composition of the product corresponds to a molar ratio of Si(C_3_H_7_O_3_)_4_–FeC_3_H_5_O_3_–HB(C_3_H_6_O_3_)_2_–C_3_H_8_O_3_–H_2_O 1.00:0.25:1.00:8.00:25.00.The resulting product was analyzed using IR spectroscopy, and AES (Si, Fe, B) (Table 5).

IR, ν/cm^−1^: 3283 (OH); 2930, 2885, (С–Н, CH_2_, CH); 1637 (Н–О–Н); 1456, 1418, 1321, 1285 (С–Н, CH_2_, CH, В–О); 1200 (Si–O–B); 1106, 1031, 1038, 993, 851, 810 (C–O, C–C, C–O–Fe, Si–O–C, Si–O–Si); 560 (Fe–O).

#### 4.3.4. Synthesis of Si-Fe-Zn-B–Gel

Silicon glycerolates in 4.5M excess of glycerol Si(C_3_H_7_O_3_)_4_·4.5C_3_H_8_O_3_ (51.48 g, 0.0638 mol), iron(III) monoglycerolate FeC_3_H_5_O_3_ (2.32 g, 0.0160 mol), zinc monoglycerolate in 6 M excess of glycerol ZnC_3_H_6_O_3_·6C_3_H_8_O_3_ (11.32 g, 0.0160 mol), and boron bisglycerolates HB(C_3_H_6_O_3_)_2_ (6.13 g, 0.0319 mol) were mixed and stirred at room temperature until a homogeneous mixture was formed. Then, H_2_O (28.75 g, 1.5950 mol) was added with vigorous stirring, and stirring continued at 70 °C until the system lost fluidity and a homogeneous light green gel was formed. The yield of the product was 100.00 g (100%). The composition of the product corresponds to a molar ratio of Si(C_3_H_7_O_3_)_4_–FeC_3_H_5_O_3_–ZnC_3_H_6_O_3_–HB(C_3_H_6_O_3_)_2_–C_3_H_8_O_3_–H_2_O 1.00:0.25:0.25:0.50:6.00:25.00.The resulting product was analyzed using IR spectroscopy and AES (Si, Fe, Zn, B) (Table 5).

IR, ν/cm^−1^: 3280 (OH); 2936, 2885, (С–Н, CH_2_, CH); 1634 (Н–О–Н); 1415, 1323, 1281 (С–Н, CH_2_, CH, B–O); 1208 (Si–O–B); 1103, 1088, 1026, 994, 923, 851, 815 (C–O, C–C, C–O–Fe, Si−O−C, Si−O−Si); 649 (Zn−O); 560 (Fe–O).

### 4.4. Separation of Dispersed (Solid) Phase and Dispersion (Liquid) Medium of Studied Hydrogel

The solid phase of studied gels was separated, similar to refs. [37,47], by exhaustive cold extraction in absolute ethanol. The solid phase obtained after extraction was analyzed by AES (content of Si, Fe, Zn, B), XRD, and IR. The quantity of Si, Fe, Zn, and B (wt.%) relative to the initial quantity in the gel (distribution between solid phase and liquid medium) were calculated based on the AES data, as well as the amount of extracted phase. The filtrate (liquid medium) was examined by AES and IR spectroscopy after evaporation to constant weight under reduced pressure. The results of the distribution of the elements in the gel are presented in Table 3.

### 4.5. Hydrogel’s Characterization

The C and H contents were measured on an automatic analyzer, PE-2400, series II CHNS-OEA 1108E (Perkin-Elmer, Shelton, CT, USA).

The IR spectra were recorded on a Perkin Elmer Spectrum Two FT-IR spectrometer (Perkin Elmer, Waltham, MA, USA) equipped with the ultra-attenuated total reflection (UATR) accessory on the diamond crystal, in the range of 400–4000 cm^−1^, with resolution of 4 cm^−1^, and number of scans 32.

Atomic emission spectrometry (AES) (Si, Fe, Zn, and B contents) was performed using an iCAP 6300 Duo optical emission spectrometer (Thermo Scientific, Waltham, MA, USA); measurement error no more than 10%.

Transmission electron microscope (TEM) micrograph was taken by a Jeol Jem 2100 (Jeol, Tokyo, Japan) equipped with an Olympus Cantaga G2 digital camera and an Oxford Inca Energy TEM 250 unit for microanalysis at an acceleration voltage of 200 kV and a current of 105 mA.

X-ray diffraction (XRD) analysis was performed on a Shimadzu XRD 700 diffractometer (Shimadzu, Tokyo, Japan) with Cu-Kα radiation (angle interval 10 < 2θ < 70). Phases were identified using the Powder Diffraction File JCPDSD-ICDD PDF2 library (set’s 1–47).

Electrospray ionization mass spectra (ESI MS) were recorded for negative ions on a qTOF maXis Impact HD ultra-high resolution mass spectrometer from Bruker Daltonik (Billerica, MA, USA) with a standard ionization source in the mass range of 50–2300 Da by injection analysis for sample solutions in acetonitrile using a syringe pump (model No. 601,553 KD Sci-entific Inc., Holliston, MA, USA); solution infusion rate of 240 µL/h.

Thermogravimetric analysis (TGA) was performed using TGA/DSC 1 (Mettler-Toledo, Greifensee, Switzerland) at a heating rate of 10 °C/min under an air atmosphere (60 mL/min). The temperature at the maximum weight loss rate (T_max_) was defined by a peak temperature of the DTG (1st derivative of the TG curve).

### 4.6. Pharmacological Studies

Experiments in vivo in laboratory animals in order to study pharmacological effects of new substances were held in accordance with Directive 2010/63/EU of the European Parliament and of the Council of 22 September 2010. The study design was approved by the Ethics Committee of the Federal State Budgetary Educational Institution of Higher Education Ural state medical university of the Ministry of Health of Russia (protocol No 11, 24 December 2021). All experiments were performed in accordance with the Guidelines for Preclinical Studies [54].

The pharmacological studies were conducted using white mice (17–21 g), Wistar rats (180–280 g), and Chinchilla rabbits (2.5–3.4 kg) obtained from the SMK Stezar nursery. Keeping the animals in the vivarium presupposed the presence of standard conditions for all animals in terms of lighting, normal diet, access to water and food, and ambient temperature of 18–20 °C.

#### 4.6.1. Toxicological Study

##### Acute Toxicity Study

Colloidal suspensions of synthesized Si-Fe-Zn-B–gel were gavaged once into the stomach through a tube in mice at a dose of 0.5 mL/10 g, and2.5 mL/100 g in rats. Groups of 10 animals were used for each dose study. Animals of the control groups, similar in size, were gavaged with the equivalent volumes of physiological solution in the same way. After 14 days, all experimental animals were observed in order to find any abnormalities such as presence of convulsions, or changes in skeletal muscle tone, color of skin and mucous membranes, breathing, or coordination. The animals were removed for the histological examination of tissues on the 15th day of the study. To conduct a morphological study, all tissue samples were subjected to standard stages of pre-preparation and embedded in paraffin, Leica RM 2245 microtome (Leica Biosystems, Nussloch, Germany) was used to prepare the histological sections. The histological sections were stained with hematoxylin-eosin. The study of the micropreparations was carried out using microscope (Olympus CX41, Tokyo, Japan) at a magnification of ×50, 100, 200, 400. The identified changes were recorded in the study protocols [52,62,63,64].

##### Skin Irritating Properties

In order to identify the skin-irritant effect, the studied Si-Fe-Zn-B–gel was applied to the Wistar rats (*n* = 10) by cutaneous application to a 4 cm^2^ skin area of the right side using a cotton gauze swab, with an exposure of 4 h. The opposite side served as a control—the application was carried out with distilled water. After 4 h, the cotton gauze swabs were removed and the skin reaction was assessed during the first 5 h after application to the skin, then once a day for 14 days [65].

##### Local Irritant Effect

Studies of the Si-Fe-Zn-B–gel’s local irritating properties using the conjunctival test method were carried out in the Chinchilla rabbits (*n* = 6). To set up the test, one drop of gel was injected with an eye pipette under the upper eyelid of the right eye at a dose of 10 mg and the same amount of distilled water was injected into the left eye (control), after which the nasolacrimal channel was lightly pressed for one minute. The results were assessed by the condition of the eye shell, the presence of hyperemia and injection of vessels of the conjunctiva and sclera, lacrimation, changes in the pupil, swelling of the eyelids, and the presence of discharge from the eyes 15, 30, 60 min and 3 h after instillation, daily for 14 days [66].

##### Skin-Resorptive Effect

The study of the skin-resorptive effect was carried out in 10 Wistar rats, which were fixed in the special cells with the tails immersed in test tubes with the studied Si-Fe-Zn-B–gel for 2/3 of the tail length. The sample was placed in a water bath with a temperature of 28–30 °C for an exposure time of 4 h. At the end of the experiment, the skin of the tails was washed up with water and soap. The animals were observed for 3 weeks to identify possible symptoms of intoxication.

##### Sensitizing Effect

The study of the sensitizing effect was carried out on Chinchilla rabbits (12 animals). Colloidal suspensions of 10% and 50% synthesized Si-Fe-Zn-B–gel in volumes of one milliliter were applied to a skin area of 4 × 4 cm on the right side and distributed evenly over the indicated area. During three days of observation, no change in skin color was detected. On the same day, a resolving dose of suspensions was applied to a similar area of skin on the opposite side for the possible manifestation of a sensitizing effect. The application was repeated on the 7, 14, 21, and 28th days of the experiment [67].

#### 4.6.2. Hemostatic Study

The study of the hemostatic effect was carried out according to the Guidelines for Preclinical Trials of Medicinal Agents [54] and in accordance with previous research [6,56,57,68,69].

The in vivo study was carried out in outbred white mice using a parenchymal bleeding model from an incised liver wound. For this purpose, 6 groups of 5 mice each were formed. The liver wound was created under Rausch ether anesthesia; the abdominal cavity was opened in all animals and a liver incision 0.5 cm long and 0.3 cm deep was made. The studied gels (Fe-Si–, Si-Fe-Zn–, Si-Fe-B–, and Si-Fe-Zn-B–gel) were applied on the cut liver wound of the experimental groups (5 rats each group). A thin layer of the administrated substance contained 50 mg/cm^2^ of gel, and after the application, the bleeding time was determined. The control animals were not treated. For the reference drug group, commercial hemostatic agent Capramine, containing 15.5% Fe_2_(SO_4_)_3_ aqueous solution, was used [58,59]. The bleeding time was determined using a stopwatch.

#### 4.6.3. Antimicrobial Study

The assessment of the antimicrobial properties (antibacterial and fungistatic activity) Fe-Si–, Si-Fe-Zn–, Si-Fe-B–, and Si-Fe-Zn-B–gel were carried out by diffusion of the antibiotic substances into agar (well method) according to the recommendations of the Clinical and Laboratory Standard Institute guidelines, as reported in a study [54,61,70]. The following strains of bacteria from the American Type Culture Collection were used (ATCC) and the National Collection of Type Cultures (NCTC): *Escherichia coli* ATCC 8739, *Staphylococcus aureus* ATCC 25923, *Staphylococcus aureus* methicillin-resistant strain (MRSA) NCTC 12493, *Pseudomonas aeruginosa* ATCC 9027, and *Streptococcus pyogenes* ATCC 19615. Two clinical strains of *Staphylococcus aureus* with different antibiotic susceptibility, including *Staphylococcus aureus* (MRSA) were also included. In addition, this method was used to study the activity of the studied glycerohydrogels relative to the strain *Candida albicans* RCPF _Y_-401/NCTC-885-653 (Table 4).

The size of the inhibition zones (in mm) of the studied samples was compared with the inhibition zone of 2.0% Fucidin cream (positive control for *S. aureus* and two clinical strains *S. aureus*), 3.0% Tetracycline ointment (positive control for *S. pyogenes*), 0.1% Gentamicine ointment (positive control for *E. coli* and *P. aeruginosa*), and 2.0% Oflomicol cream (positive control for *C. albicans*). Silicon-containing glycerohydrogel was used as a negative control (molar ratio of components Si(OCH_2_CH(OH)CH_2_OH)_4_:C_3_H_8_O_3_:H_2_O 1:6:24), without antimicrobial activity [44]. The experiment was repeated three times for each strain.

A gel weighing 50 mg was added to wells cut out in the agar-based nutrient media (diameter 10 mm); dishes with the medium were previously seeded with a lawn of microorganism cultures. Daily cultures of aerobic bacteria were used (24 h at 36 °C), along with two-day cultures of yeast-like Candida fungi (48 h at 25 °C). The optical density of the microbial suspension measured by a densitometer was 0.5 McFarland, which corresponded to 1.5·10^8^ CFU (Colony Forming Units) per mL. The inoculum dose was 1.5·10^5^ CFU per mL.

### 4.7. Statistical Analysis

For statistical analysis, a package Statistica 13.0 was used. The significance of the differences was assessed using the nonparametric Mann–Whitney and Kruskal–Wallis tests. The results are expressed as mean ± standard deviation (SD). The statistical differences were accepted as significant when *p* < 0.05.

## Figures and Tables

**Table 1:** Molar ratios of initial substances and gelation conditions.

Gel	Gel Components (mol)						Reaction Time at 70 °C (min)
	Si(C_3_H_7_O_3_)_4_	FeC_3_H_5_O_3_	ZnC_3_H_6_O_3_	HB(C_3_H_6_O_3_)_2_	C_3_H_8_O_3_	H_2_O	
Si-Fe–Gel *	1.00	0.30	−	−	3.00	24.00	90
Si-Fe-Zn–Gel	1.00	0.25	0.50	−	11.00	25.00	15
Si-Fe-B–Gel	1.00	0.25	−	1.00	8.00	25.00	540
Si-Fe-Zn-B–Gel	1.00	0.25	0.25	0.50	6.00	25.00	15

* Shown for comparison.

**Table 2:** Thermal analysis data for gel samples.

Gel	1st Decomposition Step		2nd Decomposition Step		3rd Decomposition Step		M_1000_ ** (%)
	Weight Loss (%)	T_max_ * (°C)	Weight Loss (%)	T_max_ (°C)	Weight Loss (%)	T_max_ (°C)	
Si-Fe–Gel	23.1	88	63.7	238	6.3	350	6.9
Si-Fe-Zn–Gel	19.4	84	66.5	241	7.5	346, 389	6.6
Si-Fe-B–Gel	9.8	−	77.0	240	6.3	352	6.9
Si-Fe-Zn-B–Gel	24.8	78	56.1	233	10.2	342	8.9

* T_max_ is the temperature at the maximum weight loss rate. ** M_1000_ is the residual inorganic content at 1000 °C.

**Table 3:** Distribution of elements in gel according to AES data.

Gel	Mass Content of Elements in the Dispersed Phase/Gel, Found (wt.%)				Relative Content of Elements in Dispersed Phase, Relative %			
	Si	Fe	Zn	B	Si	Fe	Zn	B
Si-Fe–Gel	1.92/2.56	1.53/1.51	−	−	75	101	−	−
Si-Fe-Zn–Gel	1.21/1.51	0.75/0.74	1.68/1.70	−	80	101	99	−
Si-Fe-B–Gel	1.23/1.58	0.73/0.75	−	0.04/0.65	78	97	−	6
Si-Fe-Zn-B–Gel	1.34/1.74	0.84/0.86	0.76/0.97	0.07/0.39	77	98	78	18

**Table 4:** Antimicrobial activity of gels.

Test Culture	Zone of Inhibition (mm) *				
	Fe-Si–Gel **	Si-Fe-Zn–Gel	Si-Fe-B–Gel	Si-Fe-Zn-B–Gel	Positive Control ***
*E. coli* ATCC 8739	0	12.0 ± 0.5	11.0 ± 0.5	20.0 ± 0.5	18.5 ± 0.5
*P. aeruginosa* ATCC 9027	0	10.0 ± 1.0	18.0 ± 1.0	22.0 ± 1.0	12.0 ± 0.5
*S. aureus* ATCC 25923	13.0 ± 1.0	13.0 ± 1.0	21.0 ± 1.0	16.0 ± 1.0	38.0 ± 0.5
*S. aureus* (MRSA) NCTC 12493	0	13.0 ± 1.5	20.0 ± 1.5	16.0 ± 1.5	41.0 ± 0.5
*S. aureus*, clinical strain	0	13.0 ± 1.5	21.5 ± 1.5	20.0 ± 1.5	41.0 ± 0.5
*S. aureus* (MRSA), clinical strain	12.5 ± 1.5	14.0 ± 1.5	22.0 ± 1.5	21.0 ± 1.5	38.0 ± 0.5
*S. pyogenes* ATCC 19615	0	21.0 ± 1.0	20.0 ± 1.0	27.0 ± 1.0	38.0 ± 0.5
*C. albicans* RCPF _Y_-401/NCTC-885-653	0	21.0 ± 1.0	25.0 ± 1.0	30.5 ± 1.0	30.0 ± 0.5

* Data are averages ± SD for three independent experiments; ** for comparison; *** 2.0% Fucidin cream for *S. aureus* and two clinical strains *S. aureus*; 3.0% Tetracycline ointment, *S. pyogenes*; 0.1% Gentamicine ointment, *E. coli* and *P. aeruginosa*; 2.0% Oflomicol cream, *C. albicans*).

**Table 5:** Mass content of elements in synthesized gels.

Element	Found (Calculated) Mass Content of Elements in the Gel (wt.%)			
	Si-Fe–Gel	Si-Fe-Zn–Gel	Si-Fe-B–Gel	Si-Fe-Zn-B–Gel
Si	2.56 (2.45)	1.51 (1.43)	1.58 (1.55)	1.74 (1.79)
Fe	1.51 (1.46)	0.74 (0.71)	0.75 (0.77)	0.86 (0.89)
Zn	−	1.70 (1.66)	−	0.97 (1.04)
B	−	−	0.65 (0.60)	0.39 (0.35)

## Data Availability

All data and materials are available upon request from the corresponding author. The data are not publicly available due to ongoing research using a part of the data.
